# Author Correction: Excitotoxic inactivation of constitutive oxidative stress detoxification pathway in neurons can be rescued by PKD1

**DOI:** 10.1038/s41467-018-03022-4

**Published:** 2018-01-30

**Authors:** Julia Pose-Utrilla, Lucía García-Guerra, Ana Del Puerto, Abraham Martín, Jerónimo Jurado-Arjona, Noelia S. De León-Reyes, Andrea Gamir-Morralla, Álvaro Sebastián-Serrano, Mónica García-Gallo, Leonor Kremer, Jens Fielitz, Christofer Ireson, Mª José Pérez-Álvarez, Isidro Ferrer, Félix Hernández, Jesús Ávila, Marina Lasa, Miguel R. Campanero, Teresa Iglesias

**Affiliations:** 1 Instituto de Investigaciones Biomédicas “Alberto Sols”, Consejo Superior de Investigaciones Científicas-Universidad Autónoma de Madrid (CSIC-UAM), C/ Arturo Duperier 4, 28029 Madrid, Spain; 2 CIBERNED, Centro de Investigación Biomédica en Red sobre Enfermedades Neurodegenerativas, Instituto de Salud Carlos III, C/ Valderrebollo, 5, 28031 Madrid, Spain; 3 Experimental Molecular Imaging (Molecular Imaging Unit), CIC biomaGUNE, Paseo Miramon, 182, 20009 San Sebastian, Spain; 4 Centro de Biología Molecular “Severo Ochoa” (CSIC-UAM), C/ Nicolas Cabrera 1, 28049 Madrid, Spain; 5 Protein Tools Unit, Centro Nacional de Biotecnologia, Consejo Superior de Investigaciones Científicas (CSIC), C/ Darwin 3, 28049 Madrid, Spain; 6 Experimental and Clinical Research Center (ECRC), Charité-Universitätsmedizin, Max-Delbrück-Center (MDC) for Molecular Medicine in the Helmholtz Association, 13125 Berlin, Germany; 7 Department of Cardiology, Heart Center Brandenburg and Medical University Brandenburg (MHB), 16321 Bernau, Germany; 8 Cancer Research Technology, EC1V 4AD London, UK; 9 Departamento de Biología (Unidad Docente Fisiología Animal), UAM, C/ Darwin 2, 28049 Madrid, Spain; 10 Instituto de Neuropatología, Hospital Universitario de Bellvitge, C/ Feixa LLarga s/n, 08907 Barcelona, Hospitalet de Llobregat Spain; 11 CIBERCV, Centro de Investigación Biomédica en Red de Enfermedades Cardiovasculares, Instituto de Salud Carlos III, 28029 Madrid, Spain; 12 Present Address: Institute of Physiological Chemistry, University Medical Center, Johannes Gutenberg University Mainz, Hanns-Dieter-Hüsch-Weg 19, 55128 Mainz, Germany; 13 Present Address: Centro Nacional de Biotecnología (CSIC), C/ Darwin 3, 28049 Madrid, Spain; 14 Present Address: Pharmidex Pharmaceutical Services, 14 Hanover Street, W1S 1YH London, UK

Correction to: *Nature Communications* 10.1038/s41467-017-02322-5, published online 22 December 2017

The original version of this Article contained an error in the spelling of the author Álvaro Sebastián-Serrano, which was incorrectly given as Álvaro Sebastián Serrano. This has now been corrected in both the PDF and HTML versions of the Article.

