# Supplementary material for: Excitotoxic inactivation of constitutive oxidative stress detoxification pathway in neurons can be rescued by PKD1
Source: Nat Commun. 2017 Dec 22;8:2275. doi: 10.1038/s41467-017-02322-5 (PMC5741635; doi:10.1038/s41467-017-02322-5)
Supplement: Supplementary file 1 — Supplementary Information [file 41467_2017_2322_MOESM1_ESM.pdf]

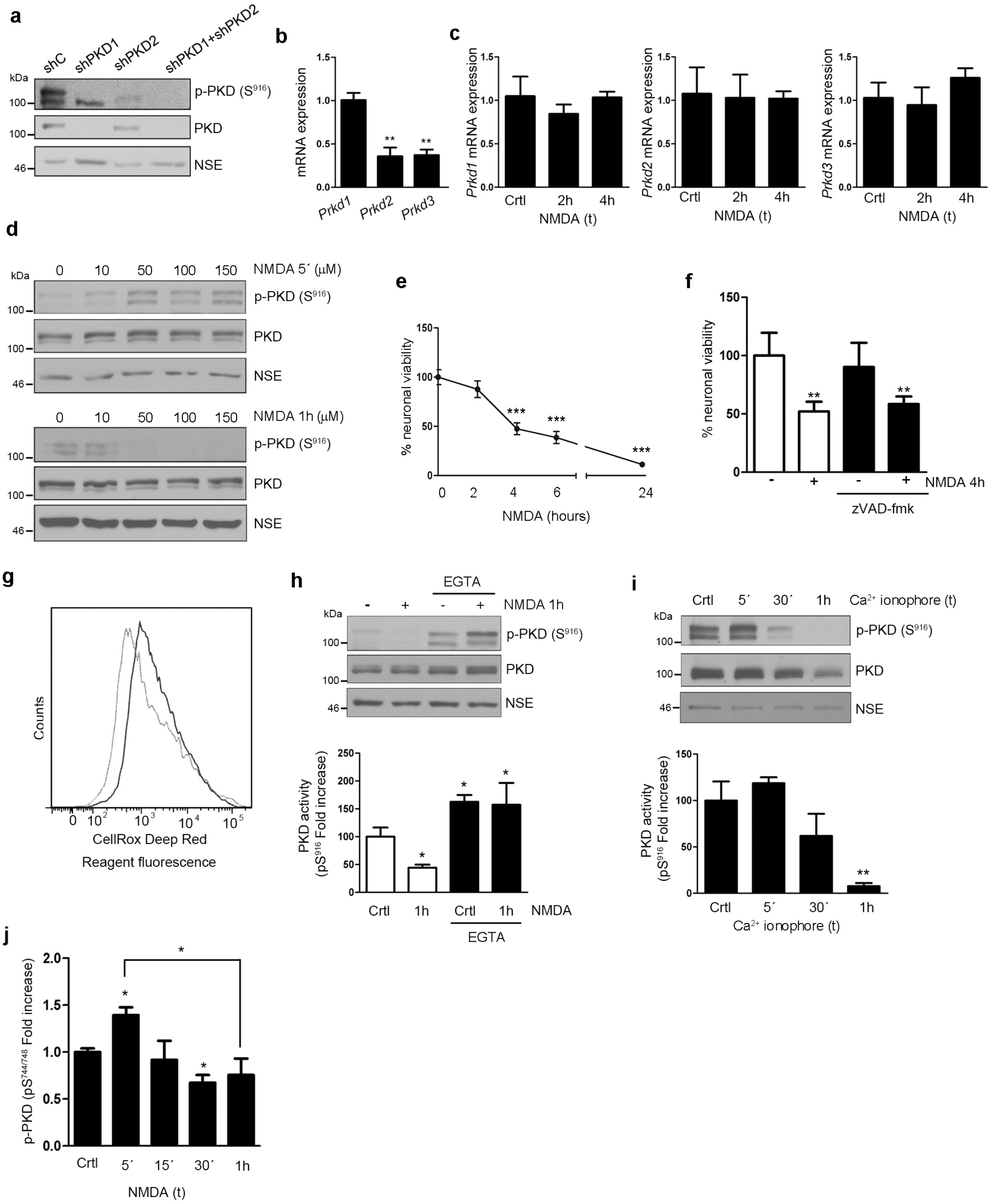

Supplementary Figure 1: PKD isoforms expression and activation in a cellular model of neuronal excitotoxicity. (a) Cultures of primary cortical neurons were transduced with lentiviral particles for control shC, shPKD1, shPKD2 or both and analysed by immunoblotting with p-PKD (S^{916}) (CST #2051) or total PKD (CST #2052) antibodies (see Supplementary Table 2). NSE was used as loading control. **(b,c)** RT-qPCR analysis of *Prkd1, Prkd2* and *Prkd3* gene expression in rat primary cortical neurons either (b) untreated or (c) stimulated with NMDA for various periods of time. Data are shown as mean$\pm$s.e.m (n=3 independent experiments) relative to (b) *Prkd1* mRNA levels or (c) untreated cells (Crtl)**. (d)** Dose-dependency of PKD activation and inactivation by NMDA treatment. Representative immunoblot of PKD activity determined by analysing p-Ser916 signal in primary cortical neurons stimulated with different NMDA doses (10-50-100-150 µM) for 5 min (top panel) or 1 h (bottom panel). NSE was used as loading control. (*n*=3 independent experiments)**. (e)** Effects of NMDA on neuronal survival. Primary cortical neurons were treated with excitotoxic concentrations of NMDA (50µM) for various periods of time and neuronal viability was measured by MTT assays. Data are expressed relative to untreated cultures as

mean±s.e.m (triplicates per condition, *n*=4 independent experiments) and analysed with two-tailed unpaired Student´s *t*-test. ****P <0.001.* **(f)** Neurons were treated with ZVAD-fmk (25 µM) for 1h and then 4h with NMDA (50µM). Neuronal viability was measured by MTT assays. Data are shown relative to untreated neurons as mean±s.e.m (triplicates per condition, *n*=3 independent experiments). **(g)** Representative flow cytometry fluorescence histograms of ROS production in cultured neurons untreated (grey line) or treated with 50µM NMDA (black line) for 1 h (*n*=3 independent experiments). **(h)** Cortical neurons were incubated for 1 h with EGTA (2mM) and then treated with 50µM NMDA for 1 h. Lysates were analysed by immunoblotting (top panels) and PKD activity was determined by Ser916 auto-phosphorylation. (Bottom panel) Quantification of p-Ser916 immunoblot signals relative to total PKD and the loading control NSE. Data are shown relative to control untreated cultures as mean±s.e.m (*n*=3 independent experiments). **(i)** (Top panels) Representative immunoblot analysis of p-Ser916 PKD, total PKD, and NSE in neurons stimulated with Ca^{2+} ionophore (A23187; 4µM) for various periods of time. (Bottom panel) Quantification of p-Ser916 values was performed as in (h) (*n*=3 independent experiments). **(j)** Quantification of immunoblot signals of PKC-mediated activatory transphosphorylation of PKD Ser744/Ser748 relative to total PKD and the loading control NSE. Data are shown relative to untreated cultures (Crtl) as mean±s.e.m (*n*=3 independent experiments). (b-j) Data were analysed with two-tailed unpaired Student´s *t*-test. **P* <0.05, ***P* <0.01, ****P* <0.001.

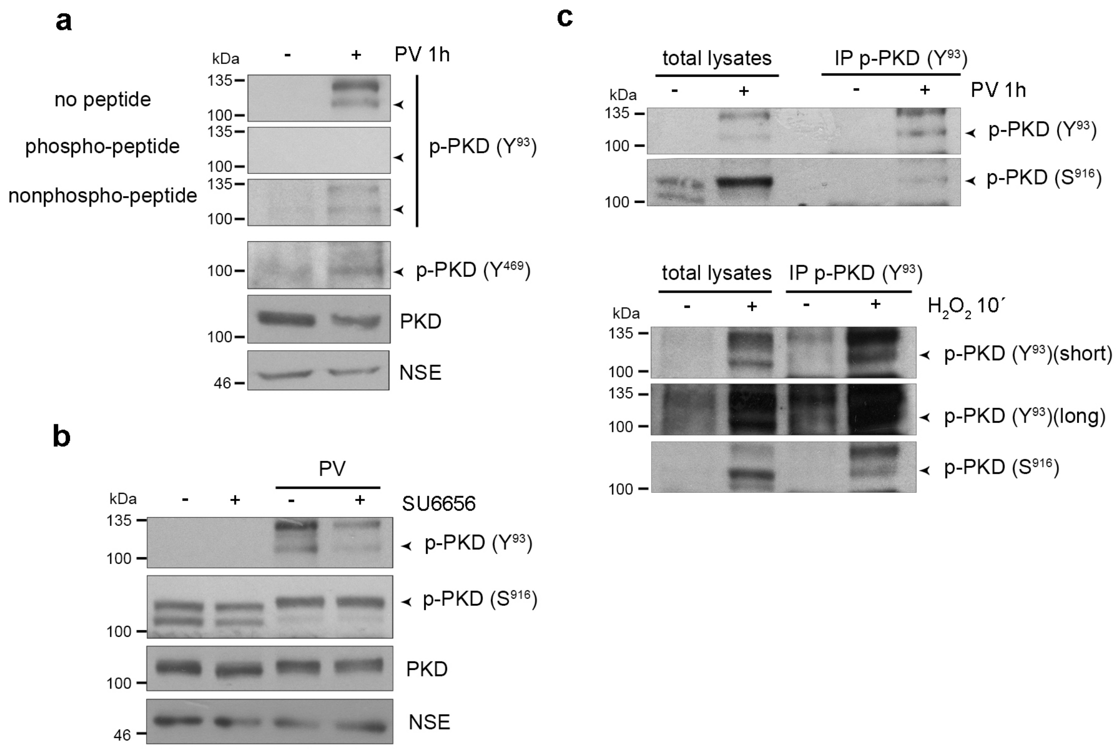

Supplementary Figure 2: Characterization of a novel monoclonal antibody (mAb) specific for PKD1 phospho-tyrosine 93 (p-Tyr93). (a) Total lysates (100 µg) from primary cortical neurons treated for 1 h with the tyrosine phosphatase inhibitor pervanadate (PV; 1 mM) were analysed by immunoblotting for tyrosine phosphorylation. The supernatant of a hybridoma clone specific for PKD1 phosphorylated at Tyr93 (see Methods) was used for immunoblot detection of p-Tyr93 before (no peptide) or after neutralization with the immunizing phospho-peptide or its non-phosphorylated version (nonphospho-peptide). The same lysates were immunoblotted for PKD Tyr469 phosphorylation, total PKD and NSE. **(b)** Primary neurons were exposed for 3 h to the Src inhibitor SU6656 (5µM), and then treated with PV (1 mM) for 1 h. Lysates were analysed by immunoblotting with antibodies to the indicated proteins. **(c)** Cortical neurons were treated with PV (1 mM) for 1 h (top panel) or stimulated with H_2O_2 (1 mM) for 10 min (bottom panel). Total lysates and PKD p-Tyr93 immunoprecipitates were analysed by immunoblotting with anti p-Tyr93 and p-Ser916 antibodies. Short and long exposure images of PKD p-Tyr93 signal are included. Representative immunoblots are shown.

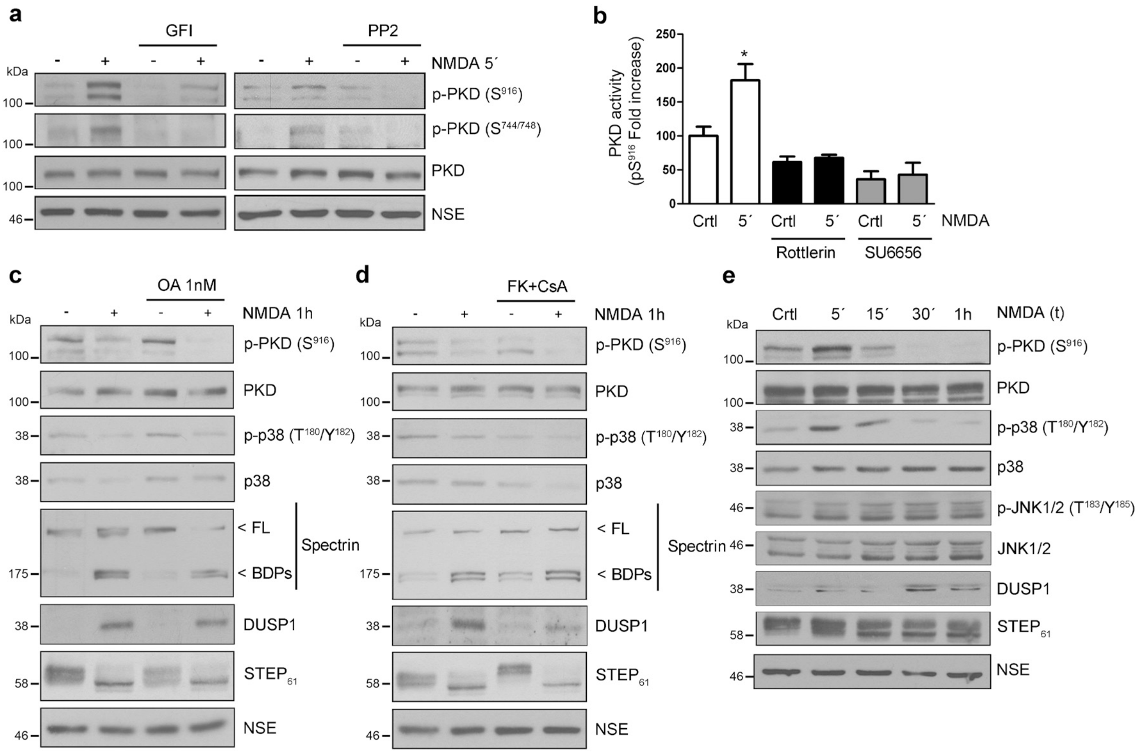

Supplementary Figure 3: Excitotoxic PKD early activation relies on PKC and Src family of kinases, whereas its dephosphorylation is independent of PP2A and PP2B and resembles that of p38. (a) Primary cortical neurons were incubated for 1 h with general inhibitors for PKC or Src family of kinases, GFI (3.5μM) or PP2 (5μM), respectively, and then treated with NMDA for 5 min. **(b)** Quantification of pSer916 immunoblot from lysates of cortical neurons incubated for 1 h with the PKC δ inhibitor Rottlerin (5 μM) or the Src inhibitor SU6656 (5μM), and then treated with NMDA for 5 min. The pSer916 signal was normalized to that of total PKD and NSE, and each value was represented relative to that of untreated cultures (Crtl). Quantification data are shown as mean±s.e.m (n=3 independent experiments) and analysed with two-tailed unpaired Student´s t-test. $^{*}P<0.05$. **(c-e)** Cultures of primary cortical neurons were incubated for 1 h with NMDA in the absence or presence of (**c**) 1nM OA, as inhibitor of PP2A, or (**d**) 200ng/ml FK506 in combination with 100ng/ml cyclosporine A (CsA), to inhibit PP2B. Total lysates were analysed by immunoblotting with the indicated antibodies. NSE was used as protein loading control. **(e)** Phosphatases activation in an *in vitro* NMDA excitotoxicity model and their effects on different kinases. Lysates from primary cortical cultures stimulated with NMDA for various periods of time were analysed by immunoblotting with the indicated antibodies. NSE was used as protein loading control. **(a-e)** Representative immunoblots of 3 independent experiments are shown.

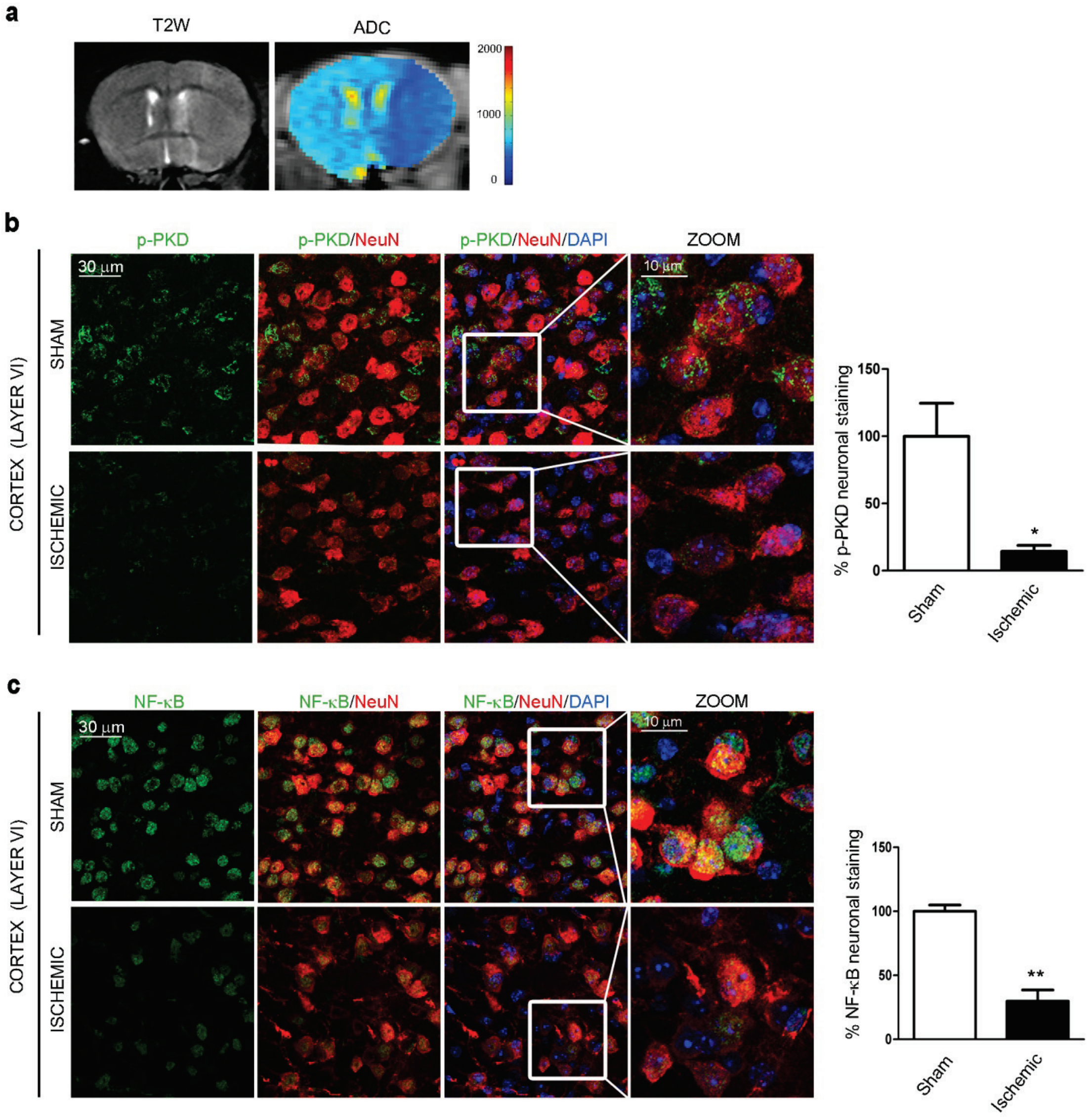

Supplementary Figure 4: Neuronal PKD/NF-κB inactivation in mouse ischemic brain after short reperfusion times (a-c) Wild-type mice were sham-operated or subjected to 60 min of MCAO and sacrificed 5 h after reperfusion. (a) Representative T2W and ADC Magnetic Resonance Images (MRI) showing the edema in the ischemic hemisphere 5 h or 2 h after occlusion release, respectively. The pseudo-color scale indicates maximum and minimum ADC values in $\mu m^2/s$. **(b-c)** Representative confocal microscopy images showing (b) p-Ser916 and (c) predominant nuclear localization of NF-κB staining in NeuN+ cells in brain from sham-operated animals, and the decrease in both signals on NeuN+ cells at the cortical penumbra area in ischemic brain. Zoom images from boxed regions are also shown. (Right panels) Percentage of NeuN+ cells containing p-Ser916 or nuclear NF-κB staining in the penumbra zone of MCAO-operated mice compared to the equivalent cortical region of sham-operated animals (*n*=100 neurons; n=3 sections per animal, *n*=3 animals per condition). Quantification data are shown as mean±s.e.m and analysed with two-tailed unpaired Student´s *t*-test. *P <0.05, **P <0.01.

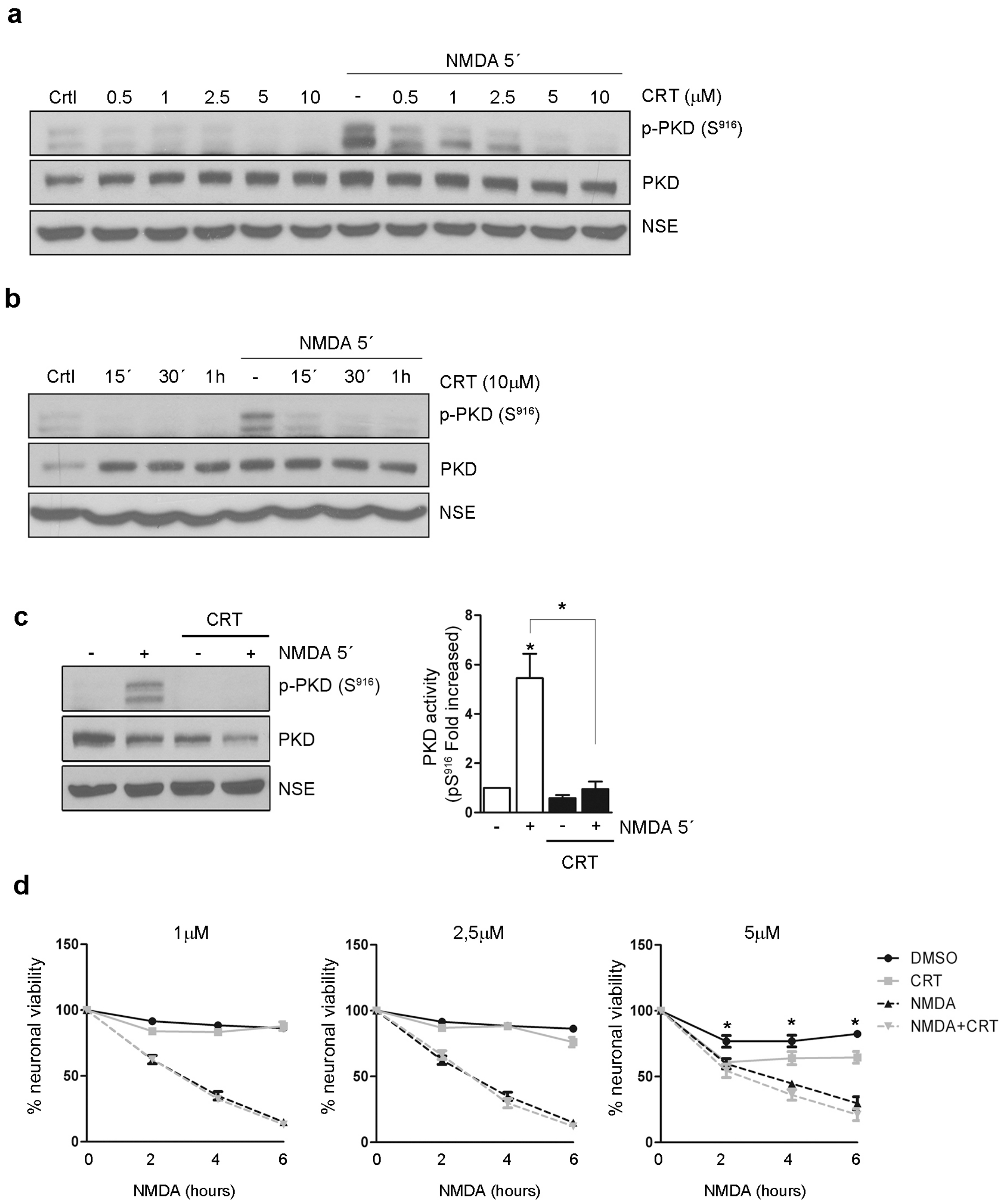

Supplementary Figure 5: Pharmacological PKD inhibition blocks basal PKD activity and NMDA-evoked PKD activation and potentiates neuronal death. **(a-b)** Representative PKD, p-PKD Ser916, and NSE immunoblot analysis from primary cortical neurons treated (a) for 1 h with the selective PKD inhibitor CRT0066101 (CRT) at different concentrations (0.5-10μM) or (b) with 10μM CRT for various periods of time before the stimulation with NMDA for 5 min (*n*=3 independent experiments). **(c)** Representative PKD, p-PKD Ser916, and NSE immunoblot analysis of primary cortical neurons incubated with 10μM CRT for 1 h before addition of NMDA for 5 min (left panel) and quantitative analysis of p-PKD Ser916 levels relative to those of total PKD and NSE expressed as fold increase over untreated cultures (right panel), (*n*=3 independent experiments)**.** **(d)** Neuronal viability was measured by MTT assays in cortical neurons treated with NMDA for 2, 4 and 6 h, in the absence or presence of different doses of CRT (1, 2.5 and 5μM). Quantification data are expressed as mean±s.e.m (triplicates per condition; *n*=3 independent experiments) and analysed with two-tailed unpaired Student´s *t*-test. **P <0.05*.

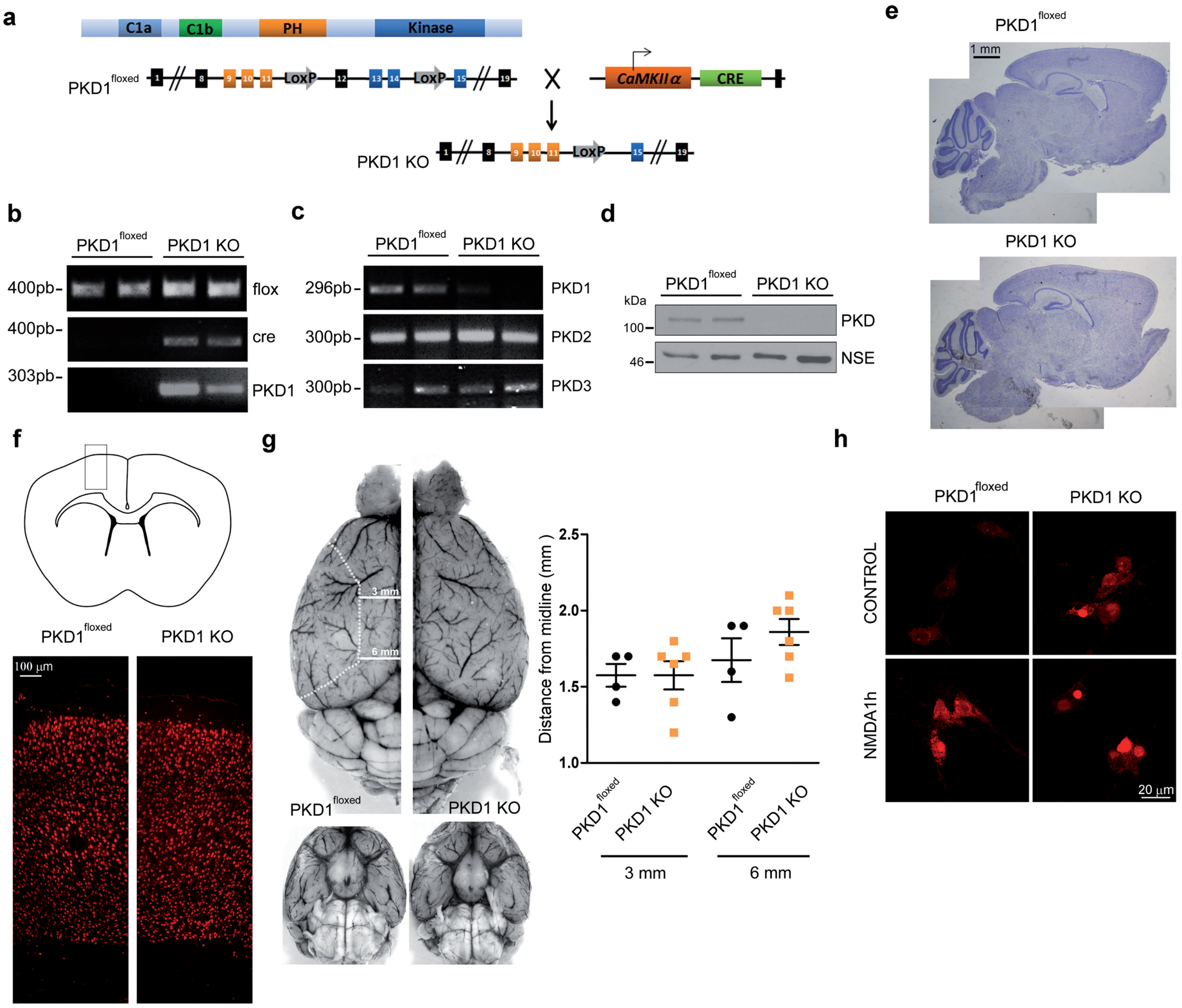
a
C1a
C1b
PH
Kinase
PKD1 floxed
LoxP
LoxP
CaMKIIα
CRE
PKD1 KO
LoxP
b
PKD1 floxed
PKD1 KO
400pb
flox
400pb
cre
303pb
PKD1
c
PKD1 floxed
PKD1 KO
296pb
PKD1
300pb
PKD2
300pb
PKD3
d
PKD1 floxed
PKD1 KO
kDa
100
PKD
46
NSE
e
PKD1 floxed
1 mm
PKD1 KO
f
PKD1 floxed
PKD1 KO
100 µm
g
3 mm
6 mm
PKD1 floxed
PKD1 KO
Distance from midline (mm)
2.5
2.0
1.5
1.0
PKD1 floxed
PKD1 KO
PKD1 floxed
PKD1 KO
3 mm
6 mm
h
PKD1 floxed
PKD1 KO
CONTROL
NMDA1h
20 µm

Supplementary Figure 6: Generation and characterization of *CaMKIIα* neuronal PKD1 KO mice. **(a)** Schematic representation of the strategy used to generate mice with *CaMKIIα*-conditional neuronal *Prkd1* deletion. **(b)** Genomic DNA PCR assessing Cre, flox and recombination in brain cortex from PKD1 KO animals and PKD1^{floxed} control littermates. **(c)** RT-PCR analysis of *Prkd1*, *Prkd2* and *Prkd3* mRNA from PKD1^{floxed} and PKD KO mice. **(d)** PKD immunoblot from cultured primary cortical neurons from PKD1^{floxed} and PKD1 KO mice. **(e-f)** Two parallel and complete series of brain slices from 2 months-old mice were stained with (e) Nissl or (f) NeuN (the area analysed by confocal microscopy is boxed in the upper brain coronal section scheme). Representative images are shown (n=3 animals per group). **(g)** Brain images of representative PKD1^{floxed} and PKD1 KO mice after Evans blue intracardial perfusion (see Methods for details). Dorsal (upper row) and ventral (lower row) view showing the absence of vascular anatomical abnormalities in both genotypes. (Right panel) Quantification of the distance between the anastomotic line (dashed line in upper row, left) and the midline at 3 and 6 mm from the frontal pole of the brain shown as mean$\pm$s.e.m (4 PKD1^{floxed} and 6 PKD1 KO animals per group). **(h)** PKD1^{floxed} and PKD1 KO cultured primary neurons unstimulated or treated with NMDAR for 1 h were stained with the oxidative stress sensitive fluorescent dye DHE to detect ROS production. Representative confocal microscopy images are shown (n=4 independent experiments).

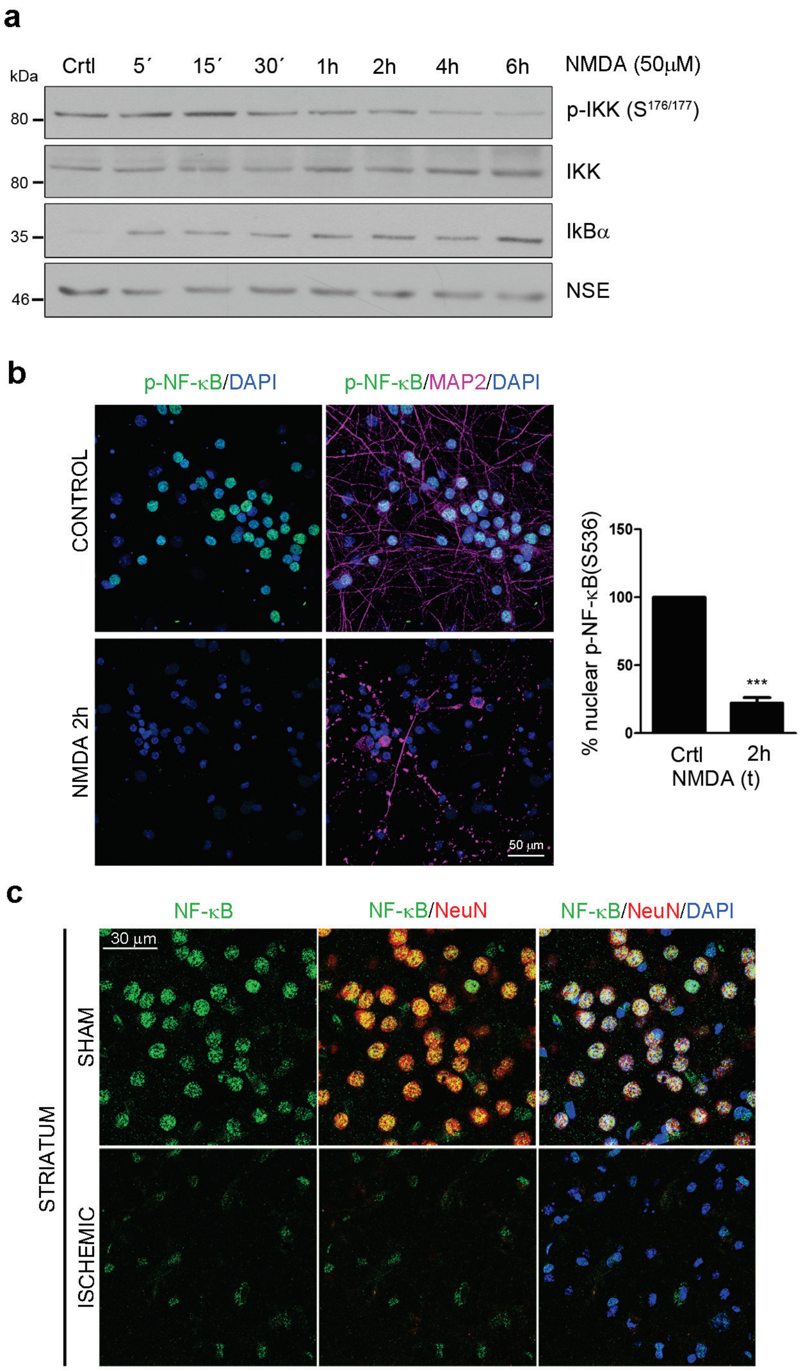

Supplementary Figure 7: IκBα accumulation and NF-κB inactivation in *in vitro* and *in vivo* models of excitotoxicity. (a) Representative immunoblots of levels and phosphorylation of IKK and IκBα from primary cortical neurons stimulated with 50μM NMDA for various periods of time (*n*=3 independent experiments). **(b)** (Left panel) Representative images of MAP2, phosphorylated active p-Ser536 at p65/NF-κB (p-NF-κB) and DAPI staining of primary cortical neurons treated with NMDA for 1 or 2 h. (Right panel) Percent of neurons bearing phosphorylated NF-κB nuclear staining before and after NMDARs overstimulation expressed relative to untreated cells (*n*=50-100 neurons per condition; *n*=3 independent experiments). Quantification data are expressed as mean±s.e.m and analysed with two-tailed unpaired Student´s *t*-test. ****P <0.001*. **(c)** Representative confocal images of NeuN, NF-κB and DAPI staining at the ischemic core in the brain striatum of sham- or MCAO-operated animals (n=3 sections per animal, *n*=3 animals per condition). See condensed nuclei in the ischemic zone.

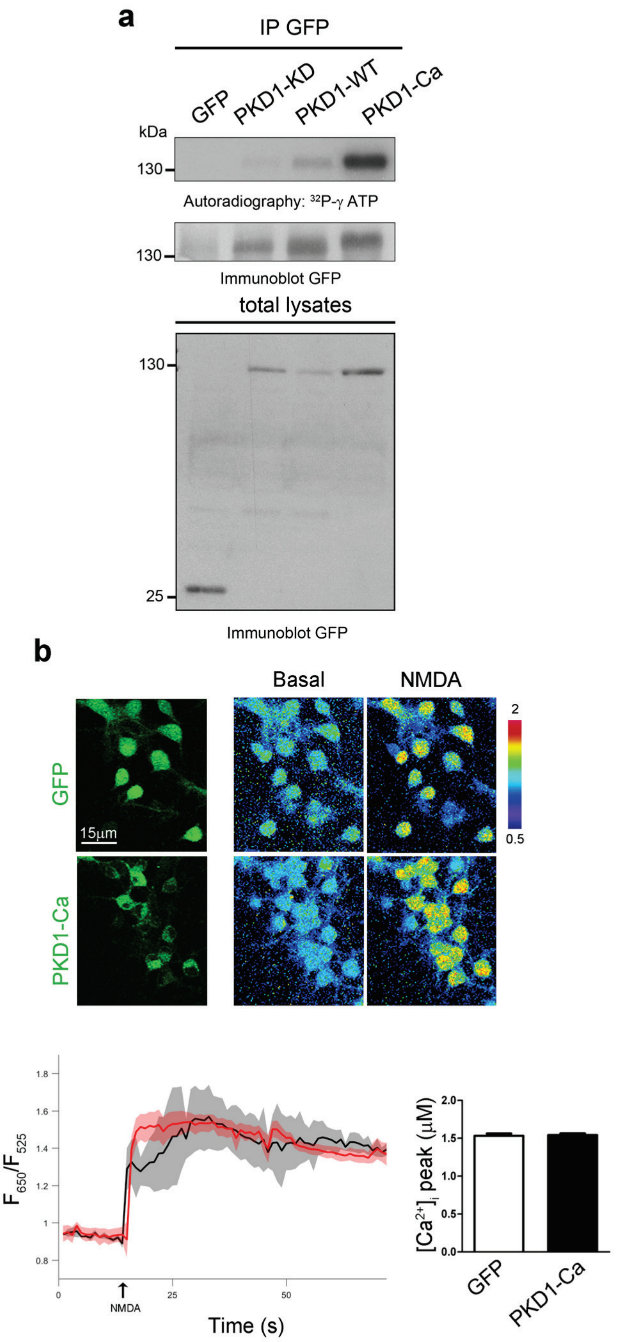

Supplementary Figure 8: PKD1 overactivation does not substantially affect Calcium influx. (a) HEK293T cells were transfected with vectors for the expression of GFP, kinase-dead mutant PKD1 **(**GFP-PKD1-KD), wild-type PKD1 (GFP-PKD1) or dephosphorylation resistant constitutively active mutant PKD1 (GFP-PKD1-Ca). Expressed GFP-fused proteins were immunoprecipitated with anti-GFP antibodies and subjected to an *in vitro* kinase assay followed by SDS-PAGE. After membrane transfer, autoradiography (top panel) and anti-GFP immunoblot (middle panel) of immunoprecipitates were obtained. Total lysates showed GFP levels of ectopically expressed proteins (bottom panel). (b) GFP or PKD1-Ca transduced neurons loaded with Fura red AM (5 µM) were visualized before or after NMDA stimulation. Representative images of GFP signal (left panel) and pseudocolor images representing fluorescence of Ca^{2+} are shown. The pseudo-color bar indicates minimum and maximum values of Fura Red ratio. Raw tracers of GFP (black line) or PKD1-Ca (red line) and quantification of mean maximum Ca^{2+} spike (right panel) to local application of NMDA (80 GFP and 80 PKD1-Ca neurons, n=3 independent experiments). Quantification data are expressed as mean$\pm$s.e.m and analysed with two-tailed unpaired Student´s *t*-test.

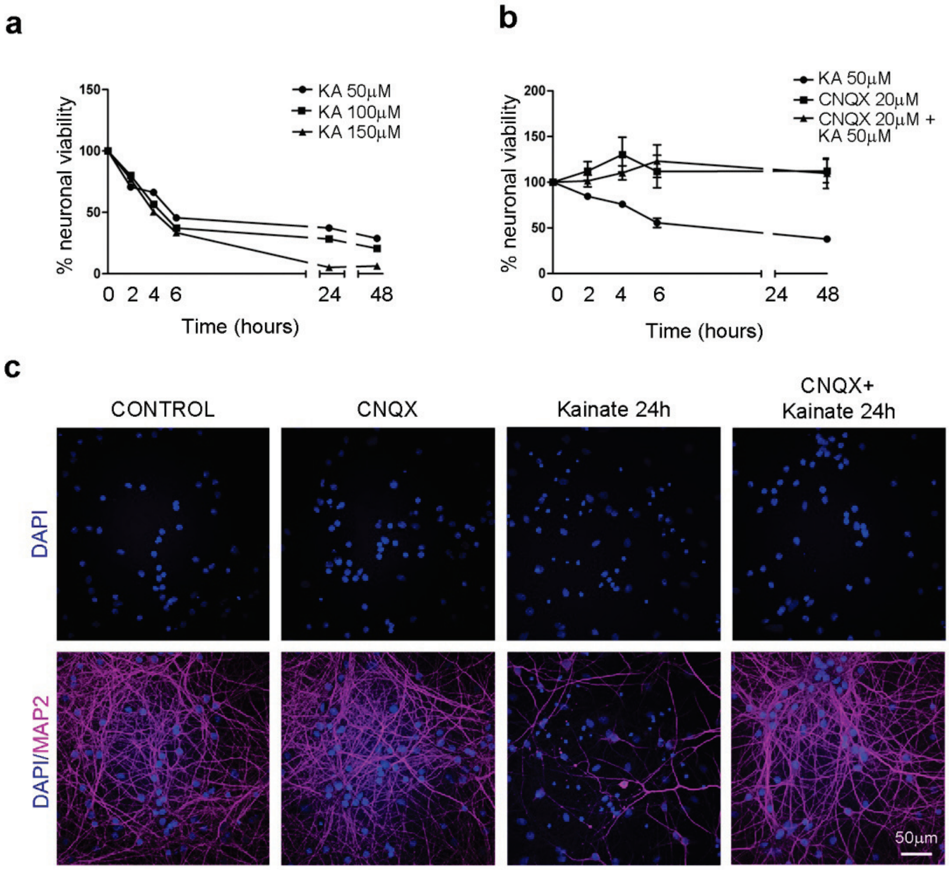

Supplementary Figure 9: ***In vitro* model of KA-induced neuronal death. (a)** Neuronal viability was measured by MTT assays in primary cortical neurons treated with different doses of KA for several periods of time (2-48 h). **(b)** Primary cortical neurons were treated with the competitive AMPA/KA receptor antagonist 6-cyano-7-nitroquinoxaline-2,3-dione (CNQX; 20μM) for 1 h prior to KA stimulation (50μM) at different time points and neuronal viability was measured by MTT assays. (a,b) Data are shown as mean±s.e.m (triplicates per condition; *n*=3 independent experiments). **(c)** Neurons cultured on coverslips were incubated with CNQX (20μM) for 1 h prior to KA (50μM) treatment during 24 h and co-stained for MAP2 and DAPI. Representative confocal microscope images of MAP2 and DAPI staining show CNQX neuroprotection from KA-induced damage (*n*=3 independent experiments).

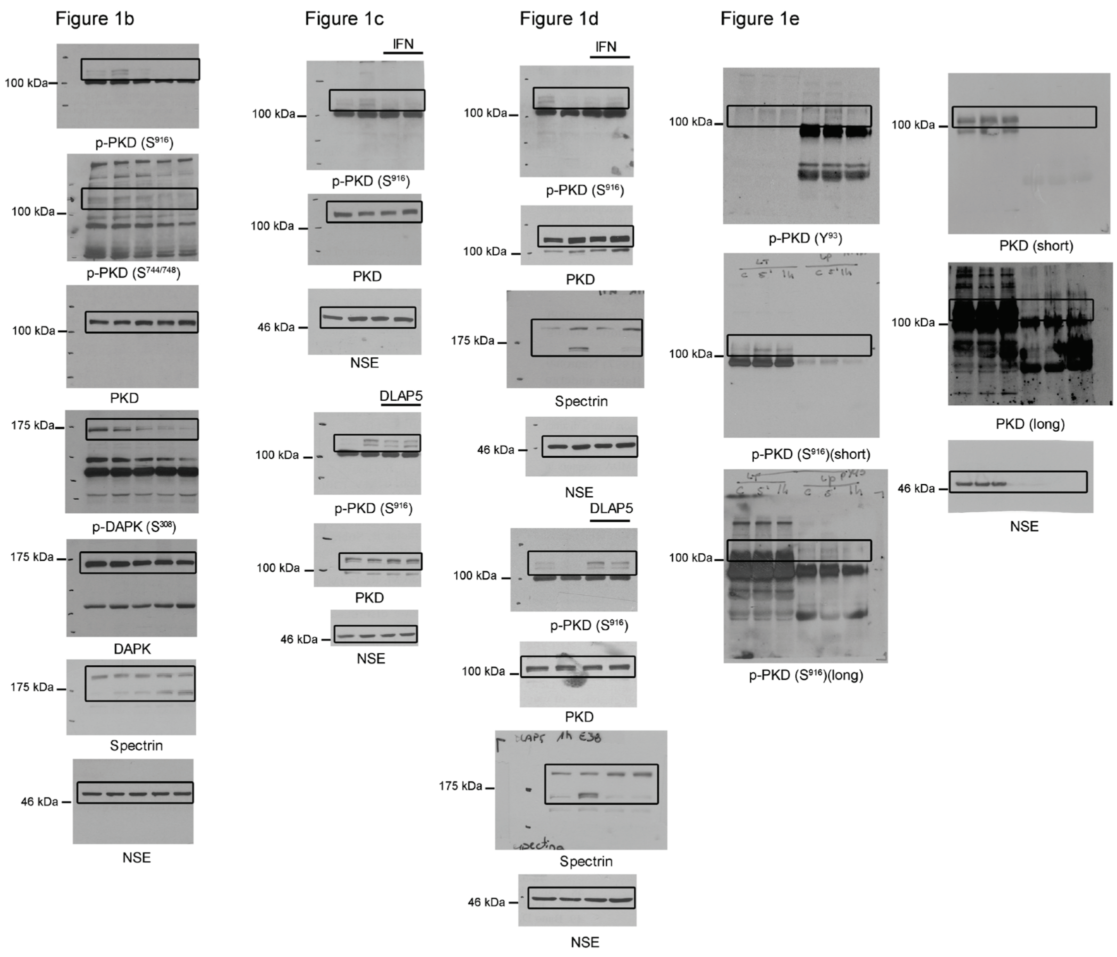

Supplementary Figure 10: Whole Western blots for Figures 1b, 1c, 1d, and 1e.

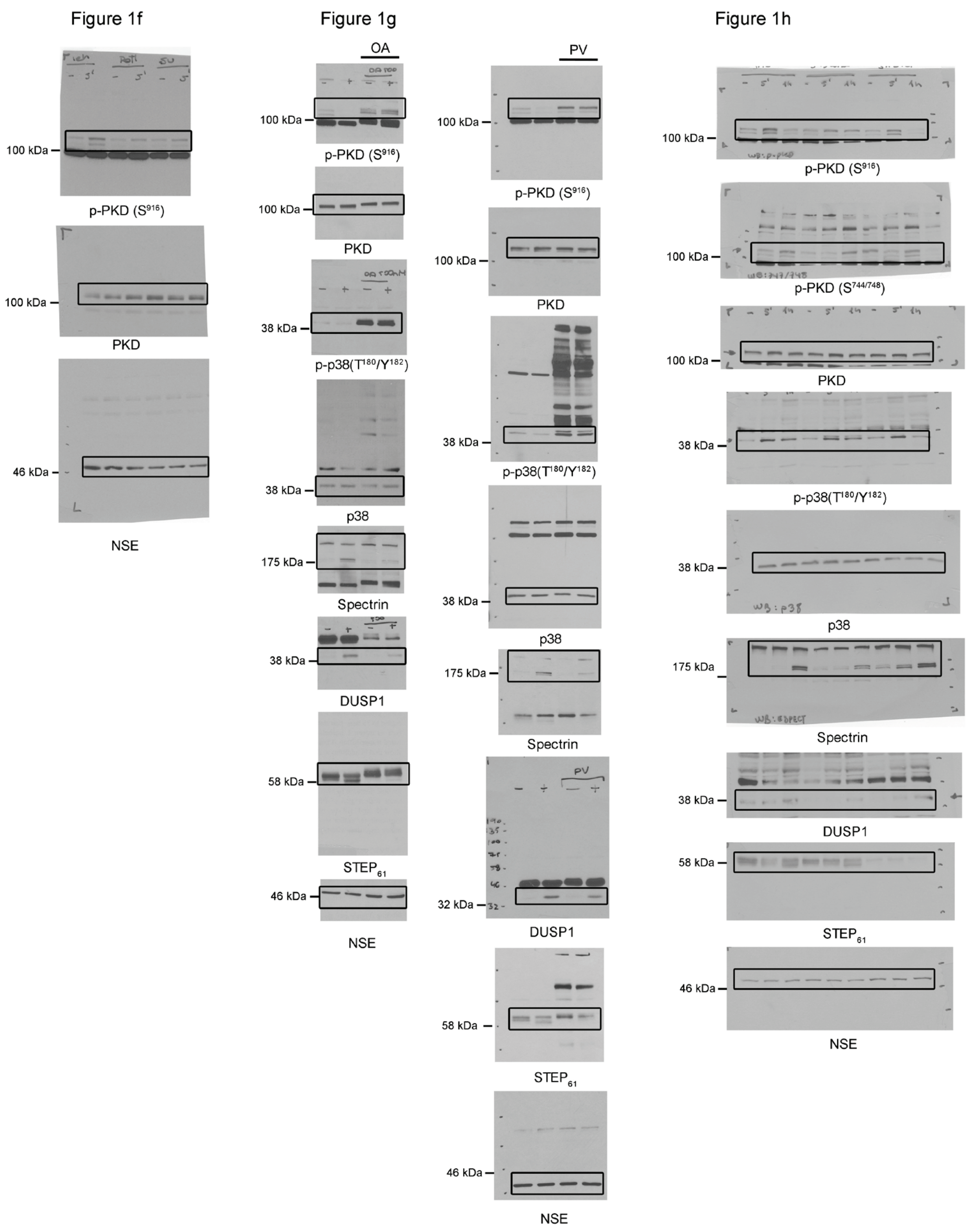

Supplementary Figure 11: Whole Western blots for Figures 1f, 1g, and 1h.

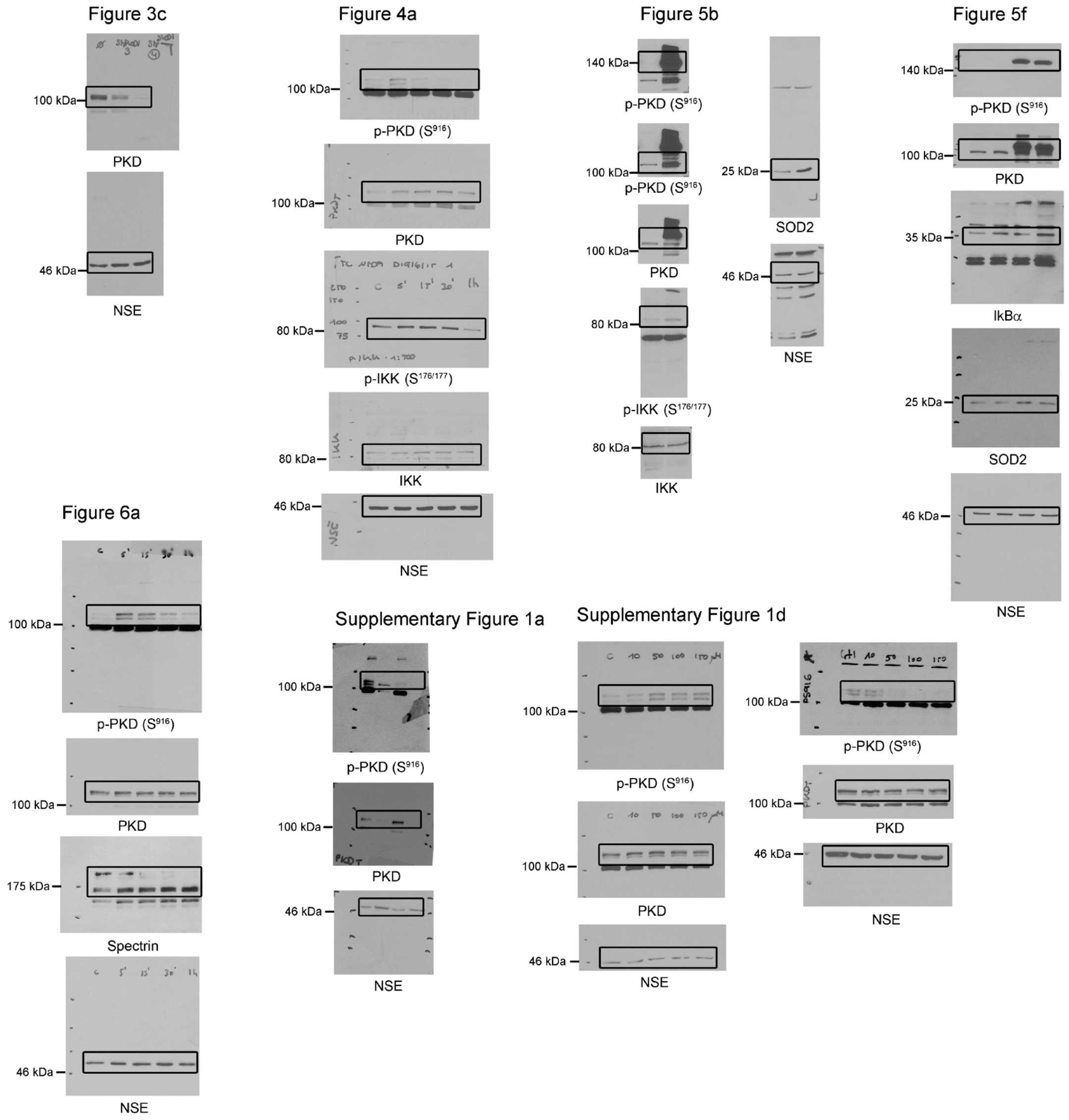

Supplementary Figure 12: Whole Western blots for Figures 3c, 4a, 5b, 5f, and 6a, and for Supplementary Figures 1a and 1d.

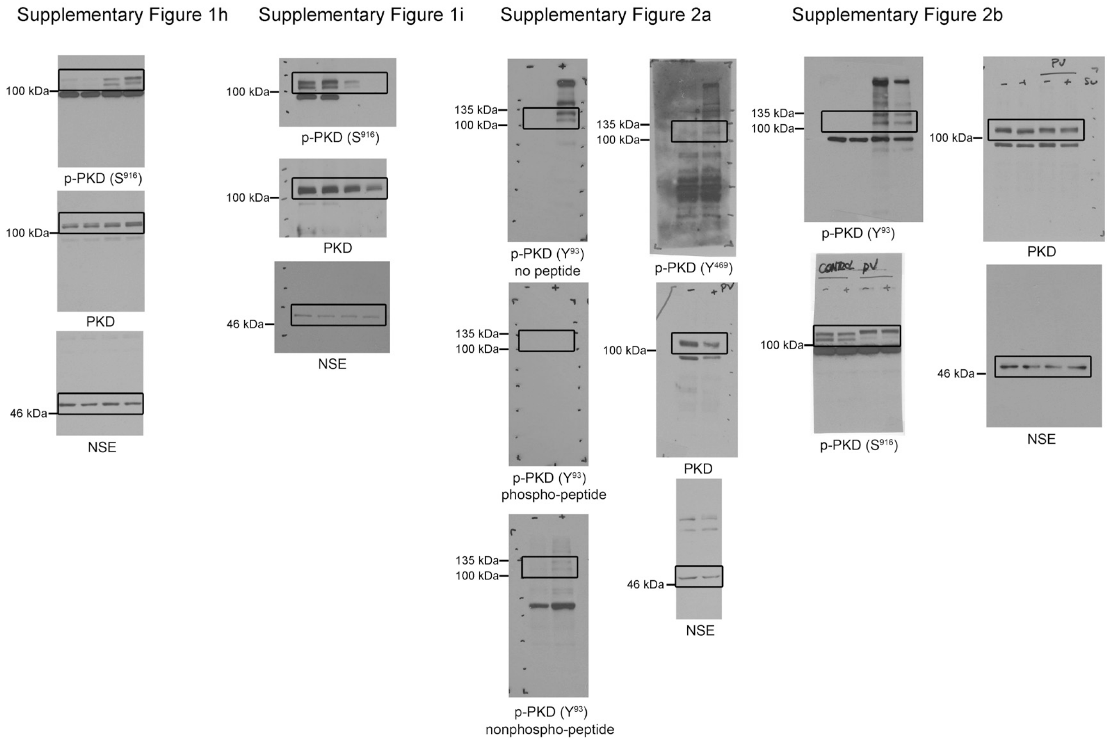

Supplementary Figure 13: Whole Western blots for Supplementary Figure 1h, Supplementary Figure 1i, Supplementary Figure 2a, and Supplementary Figure 2b.

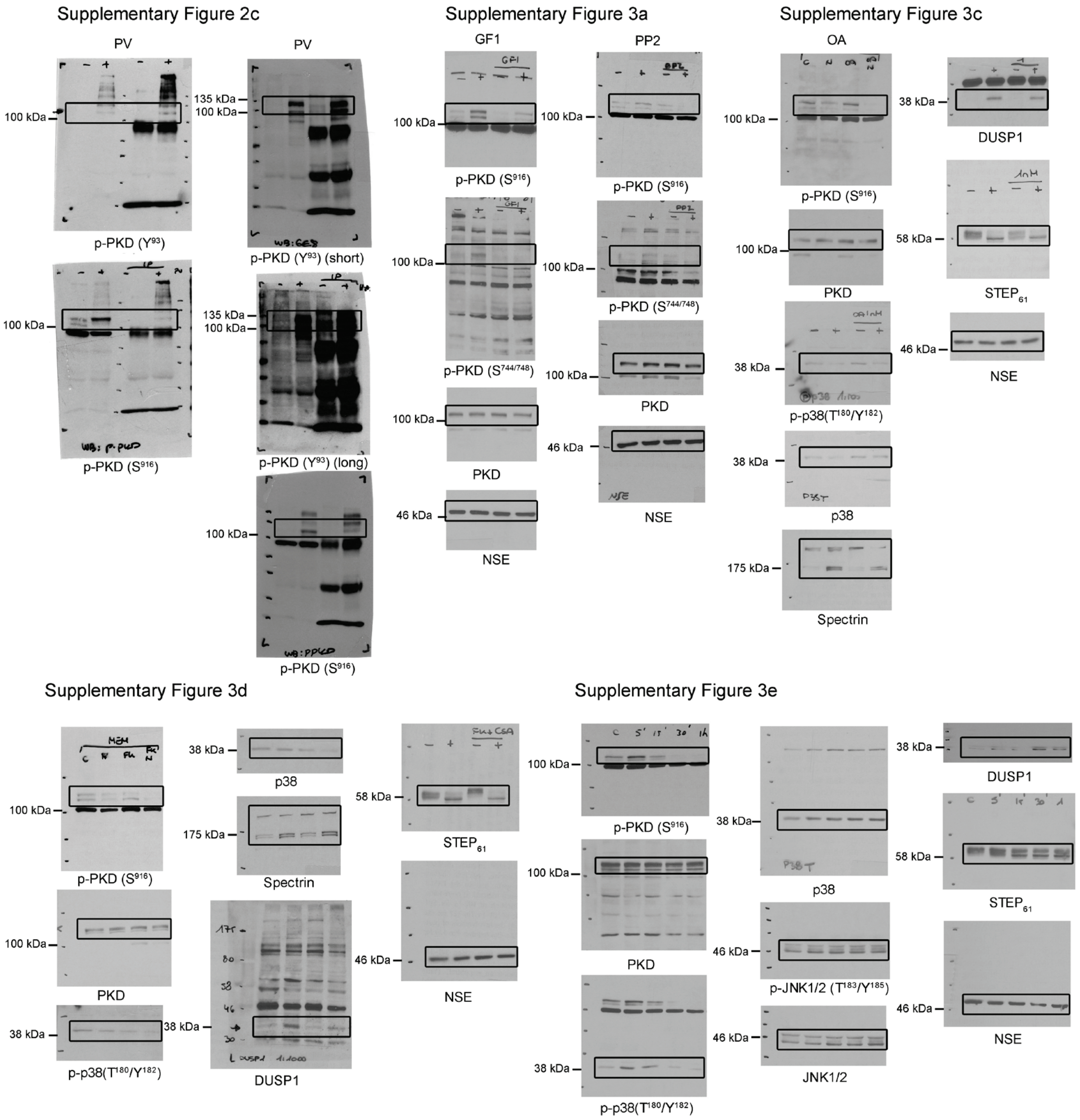

Supplementary Figure 14: Whole Western blots for Supplementary Figure 2c, Supplementary Figure 3a, Supplementary Figure 3c, Supplementary Figure 3d, and Supplementary Figure 3e.

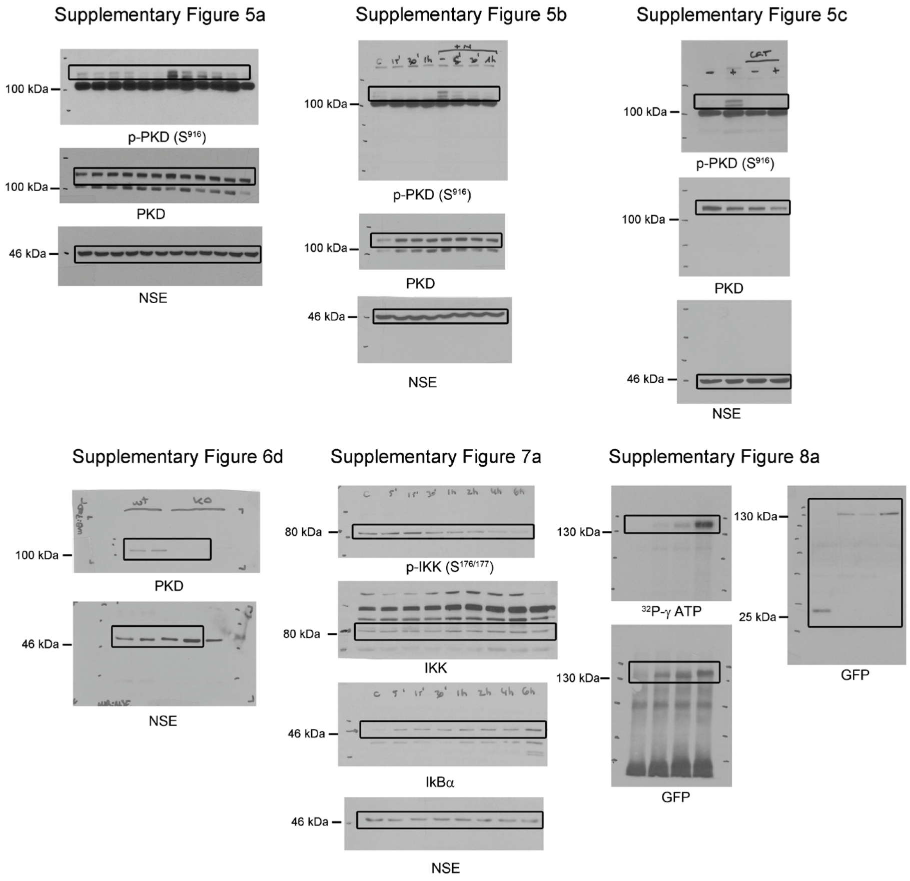

Supplementary Figure 15: Whole Western blots for Supplementary Figure 5a, Supplementary Figure 5b, Supplementary Figure 5c, Supplementary Figure 6d, and Supplementary Figure 7a, and for autoradiography and Western blots for Supplementary Figure 8a.

Supplementary Table 1. Details of the post-mortem cortex brain samples from human control individuals and stroke patients. Age (years), gender and post-mortem interval (hours) (PMI), of control subjects (C) and stroke patients (S). Post-stroke survival period and existence of recurrent strokes are also indicated.

Sample ID	Age	Gender	PMI	Post-stroke survival period	Recurrent strokes
C-1	70	M	2		_
C-2	73	M	15		_
C-3	64	M	19		_
S-1	74	M	-	7 days	None
S-2	61	M	6	> 7 days	2nd episode < 24h
S-3	89	M	3	> 7 days	None
S-4	88	F	5	> 7 days	None

Supplementary Table 2. Details of the antibodies used.

Antibody	Supplier	Catalog number	Dilution
PKD	Cell Signaling	2052	WB 1:1000
phospho-PKD-S^{916}	Cell Signaling	2051	WB 1:250<br>IMF 1:250*-1:50**
phospho-PKD-$S^{744/748}$	Cell Signaling	2054	WB 1:200
phospho-PKD-Y^{463}	Abcam	ab59415	WB 1:500
phospho-PKD1-Y^{93}, clone 6E8	T. Iglesias	-	WB Undiluited supernatant
p38	Cell Signaling	9212	WB 1:500
phospho-p38-T^{180}/Y^{182}	Cell Signaling	9211	WB 1:500
IKKβ (L570)	Cell Signaling	2678	WB 1:500
phospho-$S^{176/177}$ IKKα/β	Biorbyt	orb127876	WB 1:500
DAPK	Sigma-Aldrich	D2178	WB 1:1000
phospho-S^{308} DAPK	Sigma-Aldrich	D4941	WB 1:1000
Superoxide dismutase 2 (SOD2)	Abcam	ab13533	WB 1:1000
Microtubule associated protein 2 (MAP2)	Abcam	ab5392	IMF 1:10000
NF-kB p65 (C-20)	Santa Cruz	sc-372	IMF 1:250*-1:50**
Phospho-NF-kB p65 – S^{536}	Cell Signaling	3031	IMF 1:500
IkBα (C-21)	Santa Cruz	sc-371	WB 1:500
DUSP1	Santa Cruz	sc-370	WB 1:500
NSE	Millipore	AB951	WB 1:10000
NeuN, clone A60	Millipore	MAB377	IMF 1:1000*-1:100**
Spectrin	Millipore	MAB1622	WB 1:2000
STEP	Novus Biologicals	NB300-202SS	WB 1:1000
MDA	Jaica	1F83	IMF 1:250
GFP	Thermo Fisher Scientific Invitrogen	A6455	WB 1:1000<br>IMF 1:1000

*Cultured neurons and animal brain samples **Human brain samples
